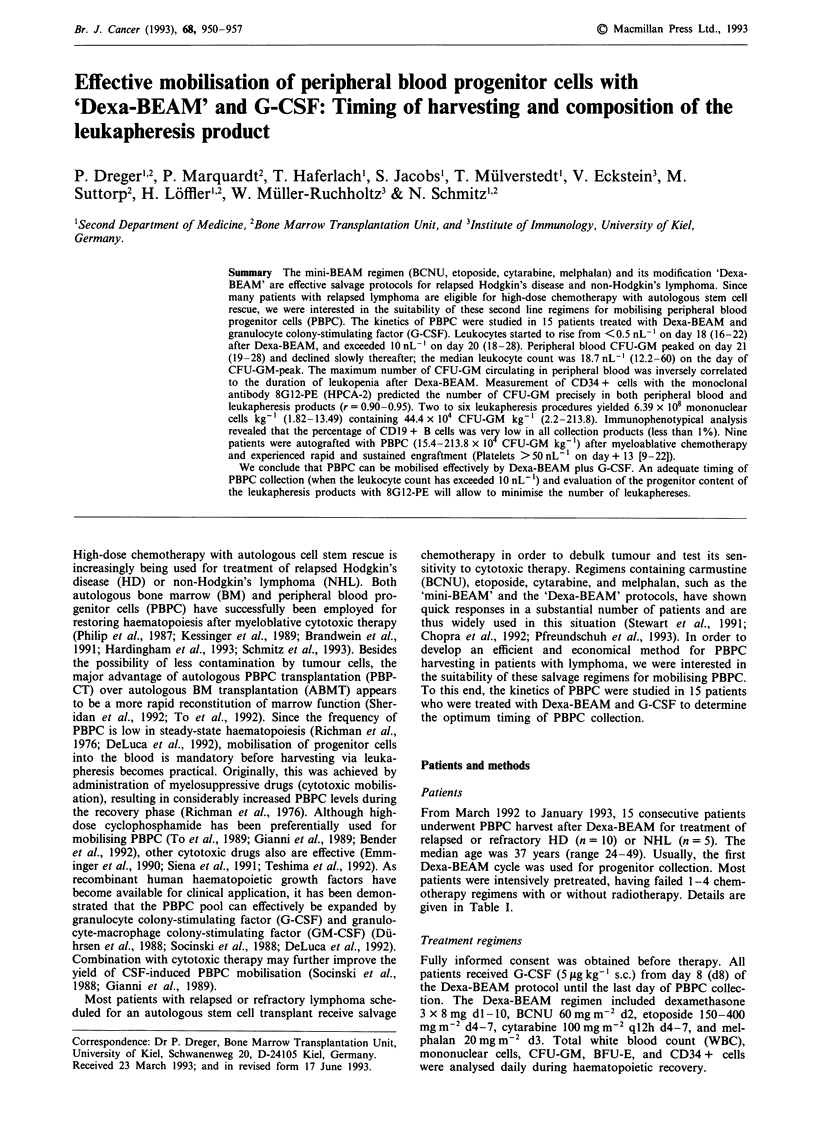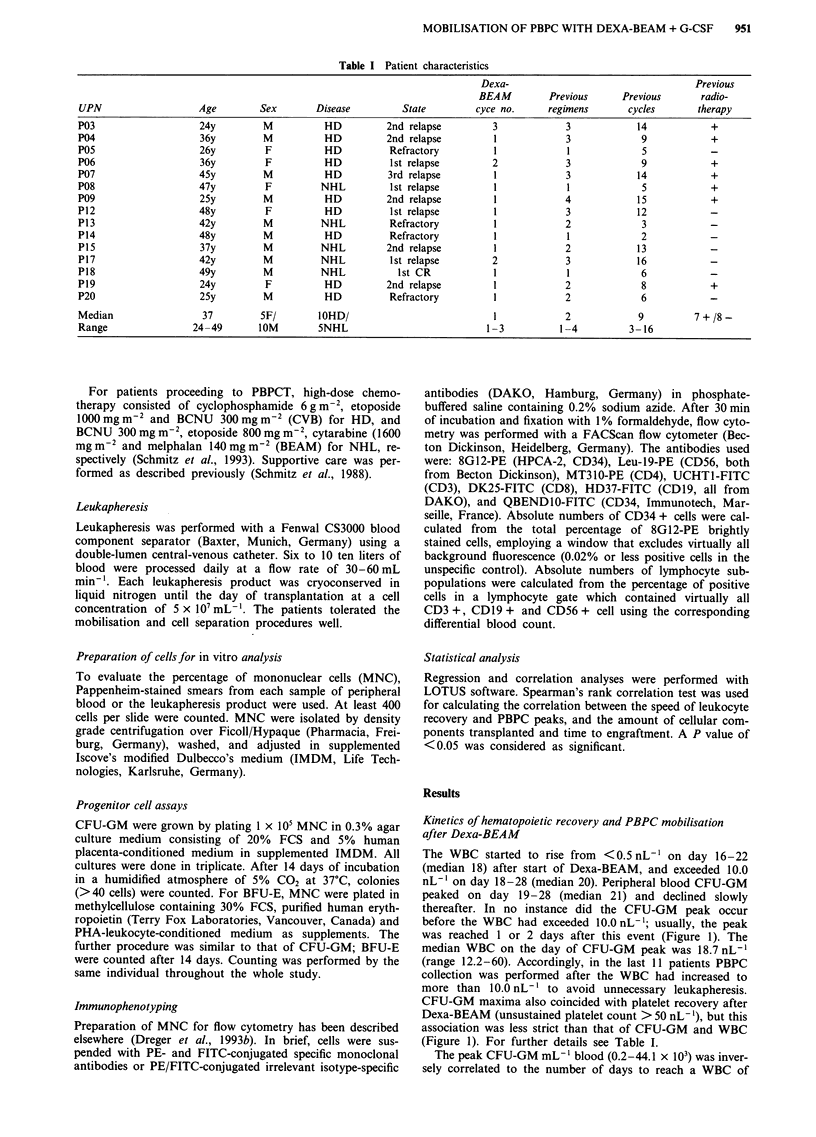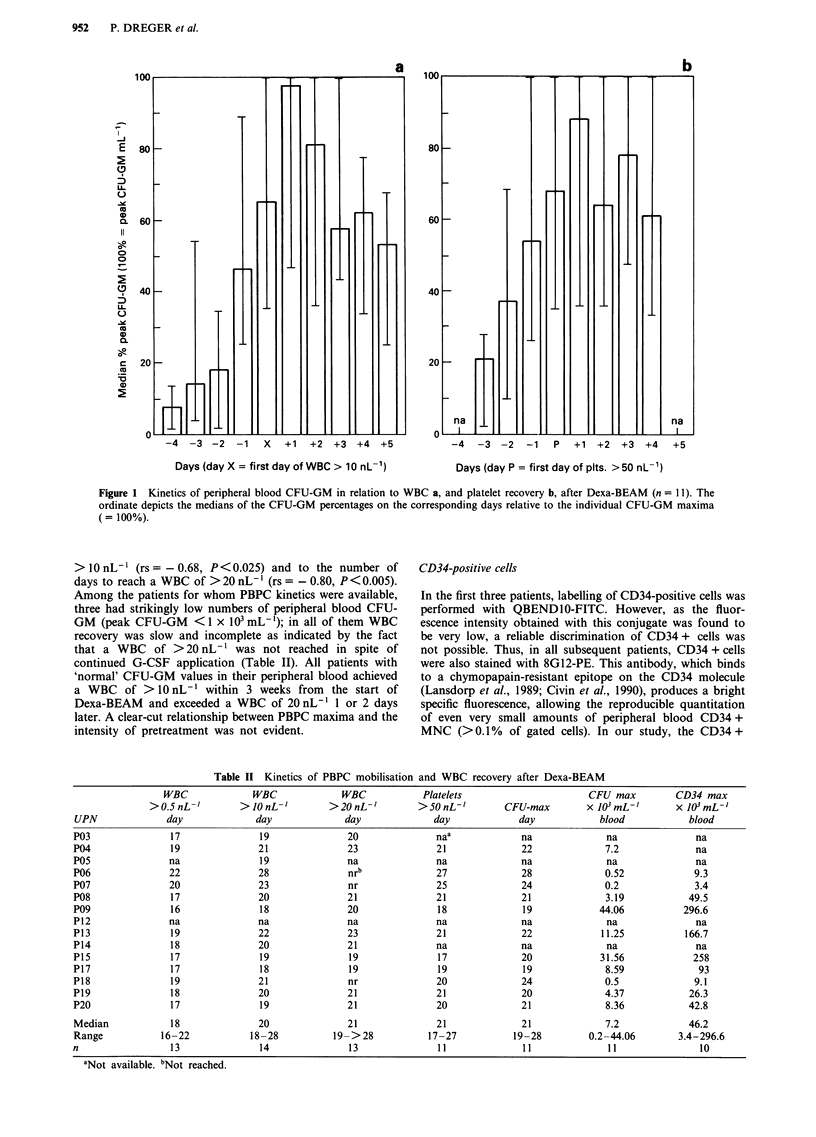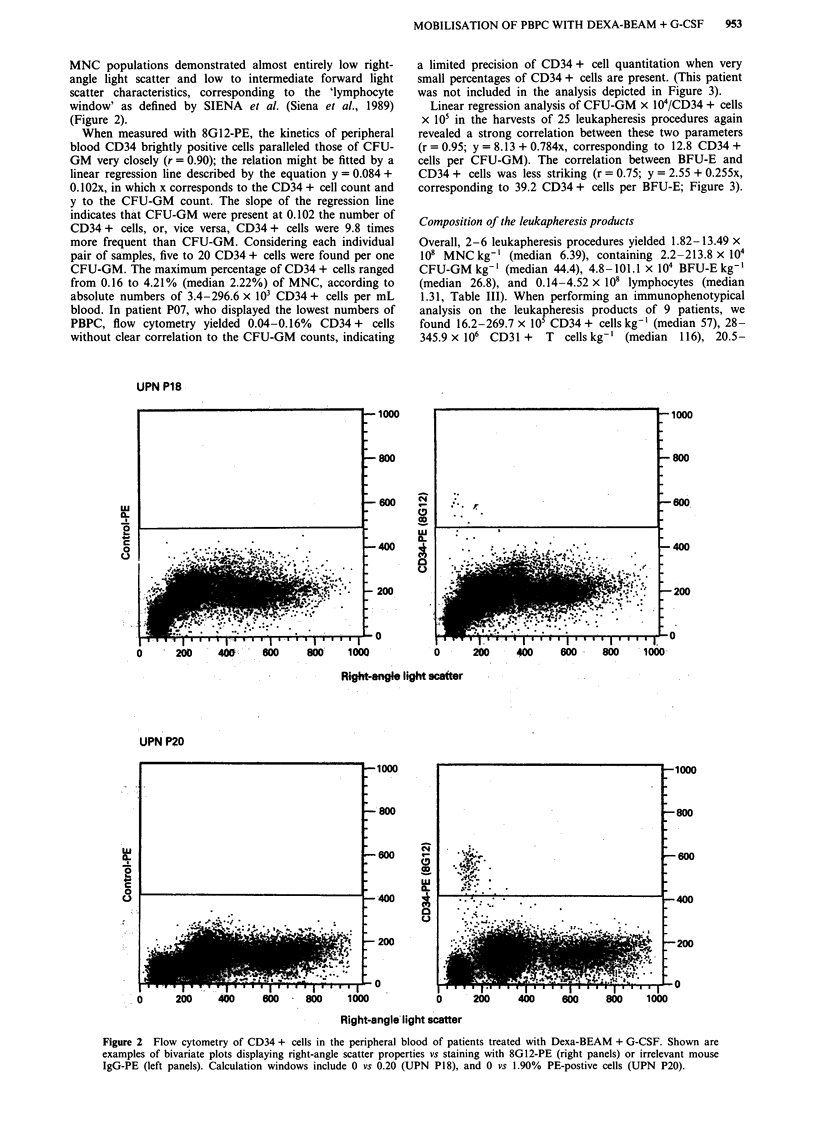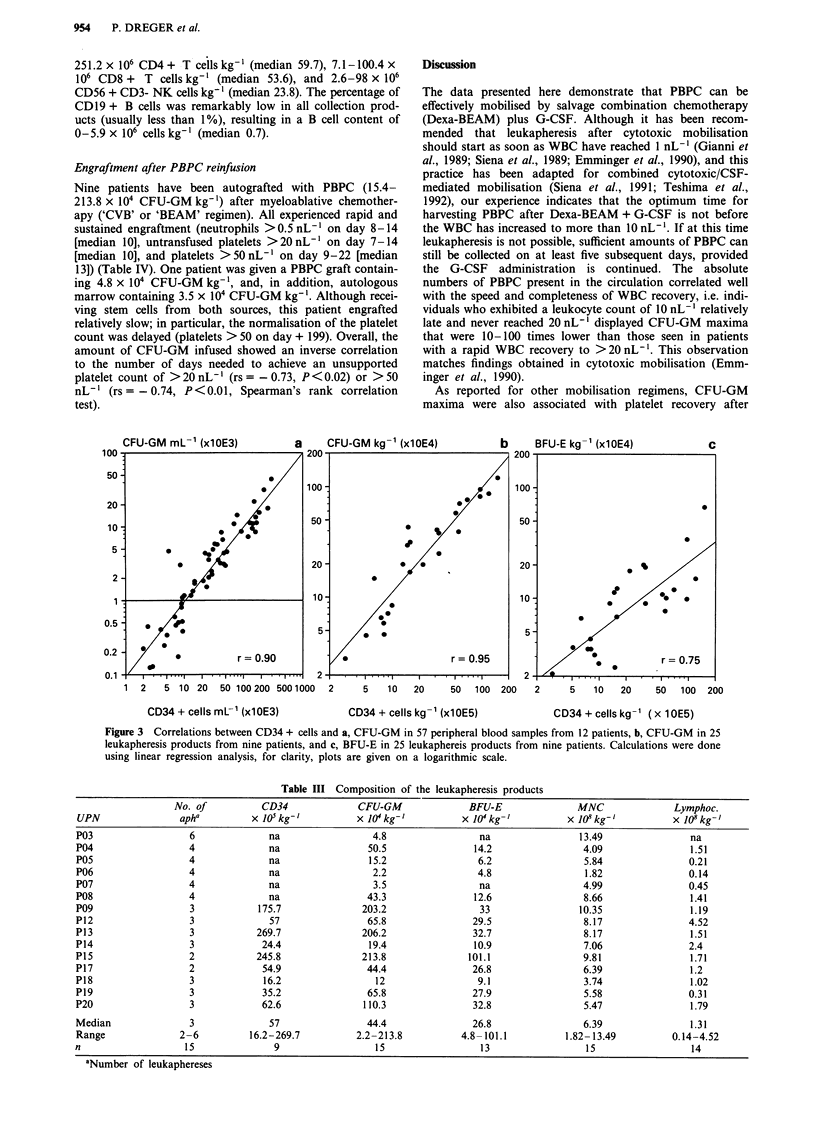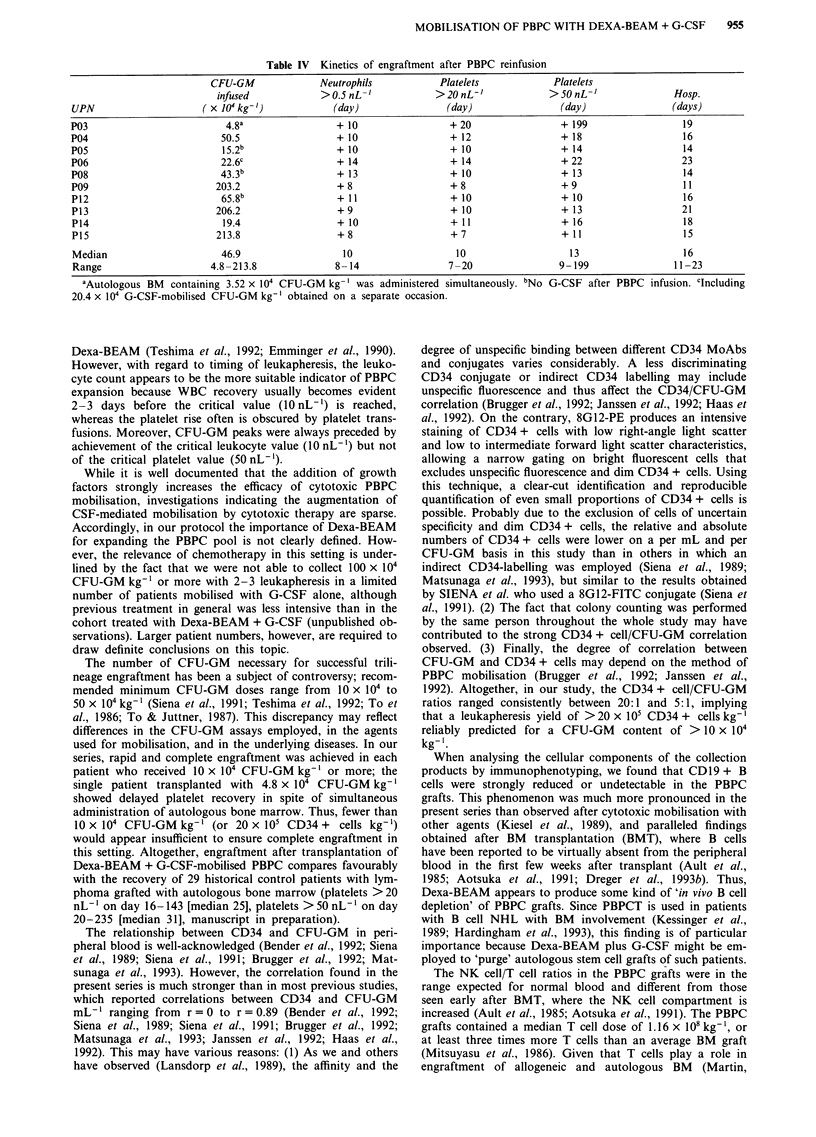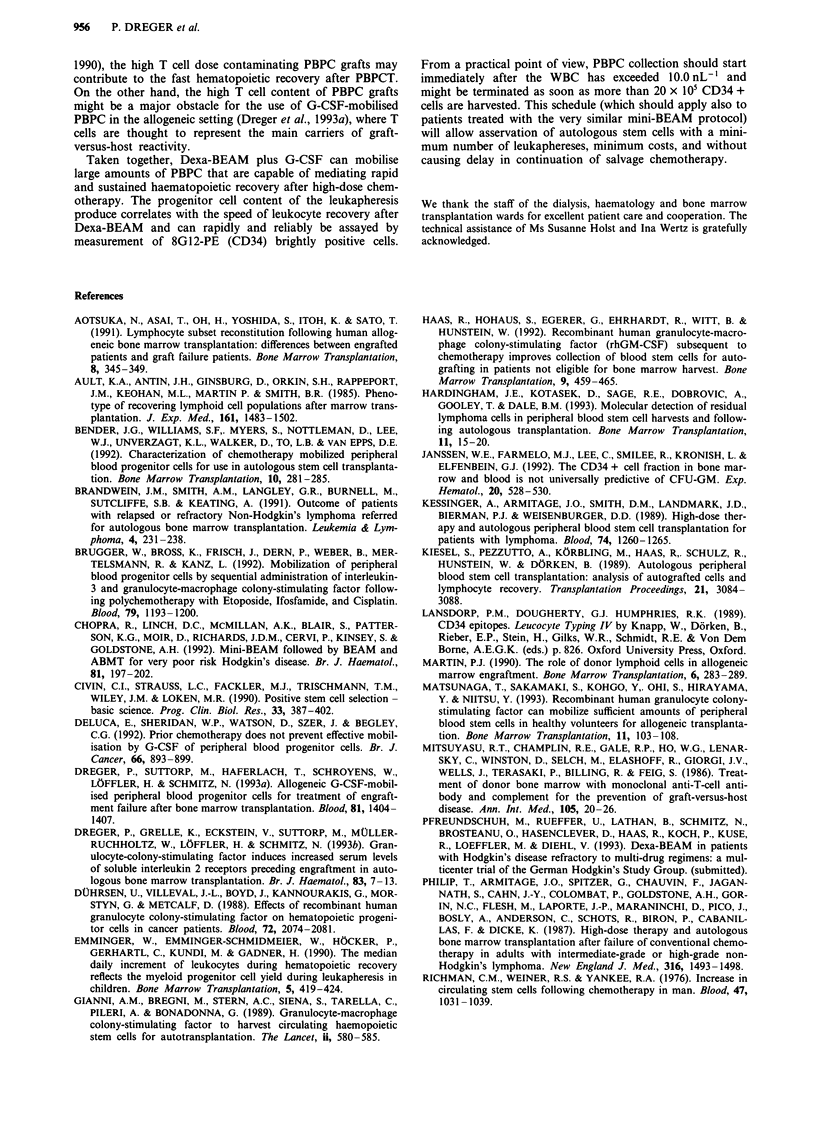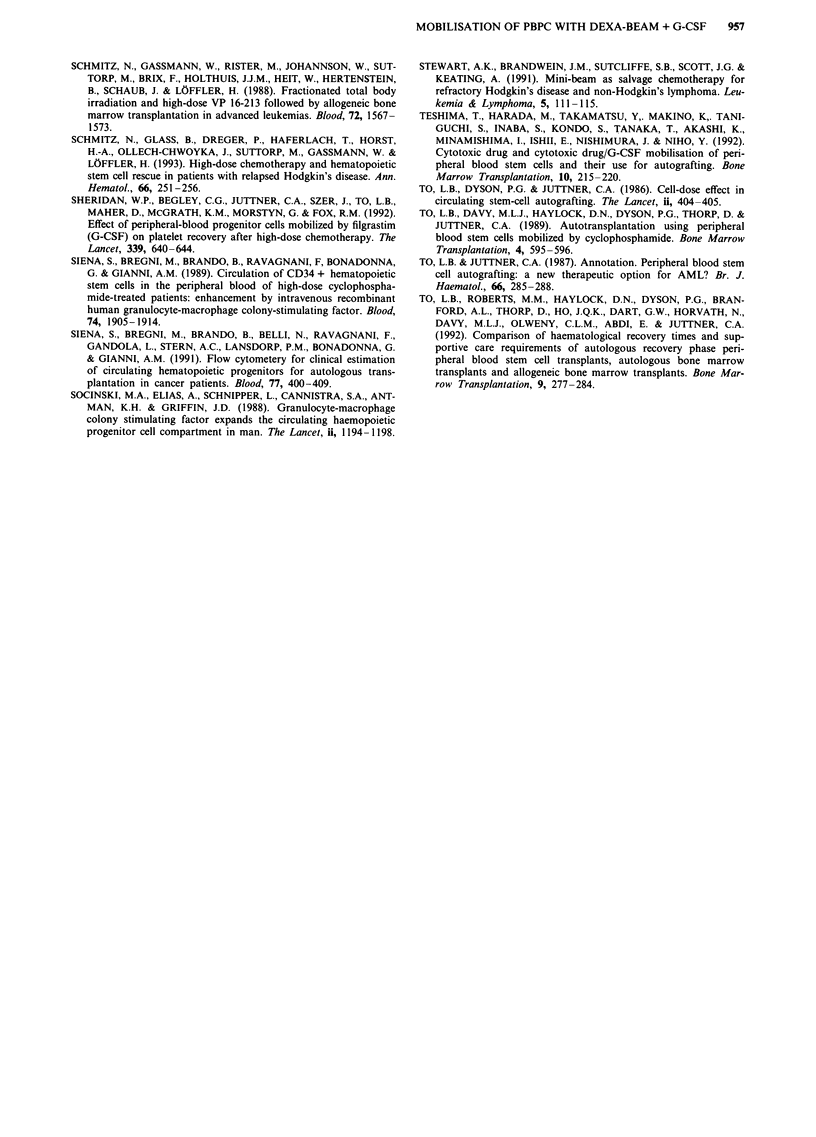# Effective mobilisation of peripheral blood progenitor cells with 'Dexa-BEAM' and G-CSF: timing of harvesting and composition of the leukapheresis product.

**DOI:** 10.1038/bjc.1993.460

**Published:** 1993-11

**Authors:** P. Dreger, P. Marquardt, T. Haferlach, S. Jacobs, T. Mülverstedt, V. Eckstein, M. Suttorp, H. Löffler, W. Müller-Ruchholtz, N. Schmitz

**Affiliations:** Second Department of Medicine, University of Kiel, Germany.

## Abstract

The mini-BEAM regimen (BCNU, etoposide, cytarabine, melphalan) and its modification 'Dexa-BEAM' are effective salvage protocols for relapsed Hodgkin's disease and non-Hodgkin's lymphoma. Since many patients with relapsed lymphoma are eligible for high-dose chemotherapy with autologous stem cell rescue, we were interested in the suitability of these second line regimens for mobilising peripheral blood progenitor cells (PBPC). The kinetics of PBPC were studied in 15 patients treated with Dexa-BEAM and granulocyte colony-stimulating factor (G-CSF). Leukocytes started to rise from < 0.5 nL-1 on day 18 (16-22) after Dexa-BEAM, and exceeded 10 nL-1 on day 20 (18-28). Peripheral blood CFU-GM peaked on day 21 (19-28) and declined slowly thereafter; the median leukocyte count was 18.7 nL-1 (12.2-60) on the day of CFU-GM-peak. The maximum number of CFU-GM circulating in peripheral blood was inversely correlated to the duration of leukopenia after Dexa-BEAM. Measurement of CD34+ cells with the monoclonal antibody 8G12-PE (HPCA-2) predicted the number of CFU-GM precisely in both peripheral blood and leukapheresis products (r = 0.90-0.95). Two to six leukapheresis procedures yielded 6.39 x 10(8) mononuclear cells kg-1 (1.82-13.49) containing 44.4 x 10(4) CFU-GM kg-1 (2.2-213.8). Immunophenotypical analysis revealed that the percentage of CD19+ B cells was very low in all collection products (less than 1%). Nine patients were autografted with PBPC (15.4-213.8 x 10(4) CFU-GM kg-1) after myeloablative chemotherapy and experienced rapid and sustained engraftment (Platelets > 50 nL-1 on day +13 [9-22]). We conclude that PBPC can be mobilised effectively by Dexa-BEAM plus G-CSF. An adequate timing of PBPC collection (when the leukocyte count has exceeded 10 nL-1) and evaluation of the progenitor content of the leukapheresis products with 8G12-PE will allow to minimise the number of leukaphereses.


Br. J. Cancer (1993), 68, 950-957 © Macmillan Press Ltd., 1993

### Effective mobilisation of peripheral blood progenitor cells with 'Dexa-BEAM' and G-CSF: Timing of harvesting and composition of the leukapheresis product

P. Dreger[1,2], P. Marquardt[2], T. Haferlach[1], S. Jacobs[1], T. Mülverstedt[1], V. Eckstein[3], M. Suttorp[2], H. Löffler[1,2], W. Müller-Ruchholtz[3] & N. Schmitz[1,2]

[1]*Second Department of Medicine,* [2]*Bone Marrow Transplantation Unit, and* [3]*Institute of Immunology, University of Kiel, Germany.*

**Summary** The mini-BEAM regimen (BCNU, etoposide, cytarabine, melphalan) and its modification 'Dexa-BEAM' are effective salvage protocols for relapsed Hodgkin's disease and non-Hodgkin's lymphoma. Since many patients with relapsed lymphoma are eligible for high-dose chemotherapy with autologous stem cell rescue, we were interested in the suitability of these second line regimens for mobilising peripheral blood progenitor cells (PBPC). The kinetics of PBPC were studied in 15 patients treated with Dexa-BEAM and granulocyte colony-stimulating factor (G-CSF). Leukocytes started to rise from $<0.5\ nL^{-1}$ on day 18 (16-22) after Dexa-BEAM, and exceeded $10\ nL^{-1}$ on day 20 (18-28). Peripheral blood CFU-GM peaked on day 21 (19-28) and declined slowly thereafter; the median leukocyte count was $18.7\ nL^{-1}$ (12.2-60) on the day of CFU-GM-peak. The maximum number of CFU-GM circulating in peripheral blood was inversely correlated to the duration of leukopenia after Dexa-BEAM. Measurement of CD34+ cells with the monoclonal antibody 8G12-PE (HPCA-2) predicted the number of CFU-GM precisely in both peripheral blood and leukapheresis products ($r = 0.90-0.95$). Two to six leukapheresis procedures yielded $6.39 \times 10^8$ mononuclear cells $kg^{-1}$ (1.82-13.49) containing $44.4 \times 10^4$ CFU-GM $kg^{-1}$ (2.2-213.8). Immunophenotypical analysis revealed that the percentage of CD19+ B cells was very low in all collection products (less than 1%). Nine patients were autografted with PBPC ($15.4-213.8 \times 10^4$ CFU-GM $kg^{-1}$) after myeloablative chemotherapy and experienced rapid and sustained engraftment (Platelets $>50\ nL^{-1}$ on day +13 [9-22]).

We conclude that PBPC can be mobilised effectively by Dexa-BEAM plus G-CSF. An adequate timing of PBPC collection (when the leukocyte count has exceeded $10\ nL^{-1}$) and evaluation of the progenitor content of the leukapheresis products with 8G12-PE will allow to minimise the number of leukaphereses.

High-dose chemotherapy with autologous cell stem rescue is increasingly being used for treatment of relapsed Hodgkin's disease (HD) or non-Hodgkin's lymphoma (NHL). Both autologous bone marrow (BM) and peripheral blood progenitor cells (PBPC) have successfully been employed for restoring haematopoiesis after myeloblative cytotoxic therapy (Philip *et al.*, 1987; Kessinger *et al.*, 1989; Brandwein *et al.*, 1991; Hardingham *et al.*, 1993; Schmitz *et al.*, 1993). Besides the possibility of less contamination by tumour cells, the major advantage of autologous PBPC transplantation (PBPCT) over autologous BM transplantation (ABMT) appears to be a more rapid reconstitution of marrow function (Sheridan *et al.*, 1992; To *et al.*, 1992). Since the frequency of PBPC is low in steady-state haematopoiesis (Richman *et al.*, 1976; DeLuca *et al.*, 1992), mobilisation of progenitor cells into the blood is mandatory before harvesting via leukapheresis becomes practical. Originally, this was achieved by administration of myelosuppressive drugs (cytotoxic mobilisation), resulting in considerably increased PBPC levels during the recovery phase (Richman *et al.*, 1976). Although high-dose cyclophosphamide has been preferentially used for mobilising PBPC (To *et al.*, 1989; Gianni *et al.*, 1989; Bender *et al.*, 1992), other cytotoxic drugs also are effective (Emminger *et al.*, 1990; Siena *et al.*, 1991; Teshima *et al.*, 1992). As recombinant human haematopoietic growth factors have become available for clinical application, it has been demonstrated that the PBPC pool can effectively be expanded by granulocyte colony-stimulating factor (G-CSF) and granulocyte-macrophage colony-stimulating factor (GM-CSF) (Dührsen *et al.*, 1988; Socinski *et al.*, 1988; DeLuca *et al.*, 1992). Combination with cytotoxic therapy may further improve the yield of CSF-induced PBPC mobilisation (Socinski *et al.*, 1988; Gianni *et al.*, 1989).

Most patients with relapsed or refractory lymphoma scheduled for an autologous stem cell transplant receive salvage chemotherapy in order to debulk tumour and test its sensitivity to cytotoxic therapy. Regimens containing carmustine (BCNU), etoposide, cytarabine, and melphalan, such as the 'mini-BEAM' and the 'Dexa-BEAM' protocols, have shown quick responses in a substantial number of patients and are thus widely used in this situation (Stewart *et al.*, 1991; Chopra *et al.*, 1992; Pfreundschuh *et al.*, 1993). In order to develop an efficient and economical method for PBPC harvesting in patients with lymphoma, we were interested in the suitability of these salvage regimens for mobilising PBPC. To this end, the kinetics of PBPC were studied in 15 patients who were treated with Dexa-BEAM and G-CSF to determine the optimum timing of PBPC collection.

Correspondence: Dr P. Dreger, Bone Marrow Transplantation Unit, University of Kiel, Schwanenweg 20, D-24105 Kiel, Germany.
Received 23 March 1993; and in revised form 17 June 1993.

### Patients and methods

### *Patients*

From March 1992 to January 1993, 15 consecutive patients underwent PBPC harvest after Dexa-BEAM for treatment of relapsed or refractory HD ($n = 10$) or NHL ($n = 5$). The median age was 37 years (range 24-49). Usually, the first Dexa-BEAM cycle was used for progenitor collection. Most patients were intensively pretreated, having failed 1-4 chemotherapy regimens with or without radiotherapy. Details are given in Table I.

### *Treatment regimens*

Fully informed consent was obtained before therapy. All patients received G-CSF ($5\ \mu g\ kg^{-1}$ s.c.) from day 8 (d8) of the Dexa-BEAM protocol until the last day of PBPC collection. The Dexa-BEAM regimen included dexamethasone $3 \times 8$ mg d1-10, BCNU $60\ mg\ m^{-2}$ d2, etoposide 150-400 $mg\ m^{-2}$ d4-7, cytarabine $100\ mg\ m^{-2}$ q12h d4-7, and melphalan $20\ mg\ m^{-2}$ d3. Total white blood count (WBC), mononuclear cells, CFU-GM, BFU-E, and CD34+ cells were analysed daily during haematopoietic recovery.

**Table I** Patient characteristics

| *UPN* | *Age* | *Sex* | *Disease* | *State* | *Dexa-BEAM cyce no.* | *Previous regimens* | *Previous cycles* | *Previous radio-therapy* |
|---|---|---|---|---|---|---|---|---|
| P03 | 24y | M | HD | 2nd relapse | 3 | 3 | 14 | + |
| P04 | 36y | M | HD | 2nd relapse | 1 | 3 | 9 | + |
| P05 | 26y | F | HD | Refractory | 1 | 1 | 5 | − |
| P06 | 36y | F | HD | 1st relapse | 2 | 3 | 9 | + |
| P07 | 45y | M | HD | 3rd relapse | 1 | 3 | 14 | + |
| P08 | 47y | F | NHL | 1st relapse | 1 | 1 | 5 | + |
| P09 | 25y | M | HD | 2nd relapse | 1 | 4 | 15 | + |
| P12 | 48y | F | HD | 1st relapse | 1 | 3 | 12 | − |
| P13 | 42y | M | NHL | Refractory | 1 | 2 | 3 | − |
| P14 | 48y | M | HD | Refractory | 1 | 1 | 2 | − |
| P15 | 37y | M | NHL | 2nd relapse | 1 | 2 | 13 | − |
| P17 | 42y | M | NHL | 1st relapse | 2 | 3 | 16 | − |
| P18 | 49y | M | NHL | 1st CR | 1 | 1 | 6 | − |
| P19 | 24y | F | HD | 2nd relapse | 1 | 2 | 8 | + |
| P20 | 25y | M | HD | Refractory | 1 | 2 | 6 | − |
| Median | 37 | 5F/ | 10HD/ | | 1 | 2 | 9 | 7 + /8 − |
| Range | 24–49 | 10M | 5NHL | | 1–3 | 1–4 | 3–16 | |

For patients proceeding to PBPCT, high-dose chemotherapy consisted of cyclophosphamide 6 g m$^{-2}$, etoposide 1000 mg m$^{-2}$ and BCNU 300 mg m$^{-2}$ (CVB) for HD, and BCNU 300 mg m$^{-2}$, etoposide 800 mg m$^{-2}$, cytarabine (1600 mg m$^{-2}$ and melphalan 140 mg m$^{-2}$ (BEAM) for NHL, respectively (Schmitz *et al.*, 1993). Supportive care was performed as described previously (Schmitz *et al.*, 1988).

*Leukapheresis*

Leukapheresis was performed with a Fenwal CS3000 blood component separator (Baxter, Munich, Germany) using a double-lumen central-venous catheter. Six to 10 ten liters of blood were processed daily at a flow rate of 30–60 mL min$^{-1}$. Each leukapheresis product was cryoconserved in liquid nitrogen until the day of transplantation at a cell concentration of $5 \times 10^7$ mL$^{-1}$. The patients tolerated the mobilisation and cell separation procedures well.

*Preparation of cells for* in vitro *analysis*

To evaluate the percentage of mononuclear cells (MNC), Pappenheim-stained smears from each sample of peripheral blood or the leukapheresis product were used. At least 400 cells per slide were counted. MNC were isolated by density grade centrifugation over Ficoll/Hypaque (Pharmacia, Freiburg, Germany), washed, and adjusted in supplemented Iscove's modified Dulbecco's medium (IMDM, Life Technologies, Karlsruhe, Germany).

*Progenitor cell assays*

CFU-GM were grown by plating $1 \times 10^5$ MNC in 0.3% agar culture medium consisting of 20% FCS and 5% human placenta-conditioned medium in supplemented IMDM. All cultures were done in triplicate. After 14 days of incubation in a humidified atmosphere of 5% $CO_2$ at 37°C, colonies (>40 cells) were counted. For BFU-E, MNC were plated in methylcellulose containing 30% FCS, purified human erythropoietin (Terry Fox Laboratories, Vancouver, Canada) and PHA-leukocyte-conditioned medium as supplements. The further procedure was similar to that of CFU-GM; BFU-E were counted after 14 days. Counting was performed by the same individual throughout the whole study.

*Immunophenotyping*

Preparation of MNC for flow cytometry has been described elsewhere (Dreger *et al.*, 1993*b*). In brief, cells were suspended with PE- and FITC-conjugated specific monoclonal antibodies or PE/FITC-conjugated irrelevant isotype-specific antibodies (DAKO, Hamburg, Germany) in phosphate-buffered saline containing 0.2% sodium azide. After 30 min of incubation and fixation with 1% formaldehyde, flow cytometry was performed with a FACScan flow cytometer (Becton Dickinson, Heidelberg, Germany). The antibodies used were: 8G12-PE (HPCA-2, CD34), Leu-19-PE (CD56, both from Becton Dickinson), MT310-PE (CD4), UCHT1-FITC (CD3), DK25-FITC (CD8), HD37-FITC (CD19, all from DAKO), and QBEND10-FITC (CD34, Immunotech, Marseille, France). Absolute numbers of CD34 + cells were calculated from the total percentage of 8G12-PE brightly stained cells, employing a window that excludes virtually all background fluorescence (0.02% or less positive cells in the unspecific control). Absolute numbers of lymphocyte subpopulations were calculated from the percentage of positive cells in a lymphocyte gate which contained virtually all CD3 +, CD19 + and CD56 + cell using the corresponding differential blood count.

*Statistical analysis*

Regression and correlation analyses were performed with LOTUS software. Spearman's rank correlation test was used for calculating the correlation between the speed of leukocyte recovery and PBPC peaks, and the amount of cellular components transplanted and time to engraftment. A $P$ value of $<0.05$ was considered as significant.

### Results

*Kinetics of hematopoietic recovery and PBPC mobilisation after Dexa-BEAM*

The WBC started to rise from $<0.5$ nL$^{-1}$ on day 16–22 (median 18) after start of Dexa-BEAM, and exceeded 10.0 nL$^{-1}$ on day 18–28 (median 20). Peripheral blood CFU-GM peaked on day 19–28 (median 21) and declined slowly thereafter. In no instance did the CFU-GM peak occur before the WBC had exceeded 10.0 nL$^{-1}$; usually, the peak was reached 1 or 2 days after this event (Figure 1). The median WBC on the day of CFU-GM peak was 18.7 nL$^{-1}$ (range 12.2–60). Accordingly, in the last 11 patients PBPC collection was performed after the WBC had increased to more than 10.0 nL$^{-1}$ to avoid unnecessary leukapheresis. CFU-GM maxima also coincided with platelet recovery after Dexa-BEAM (unsustained platelet count $>50$ nL$^{-1}$), but this association was less strict than that of CFU-GM and WBC (Figure 1). For further details see Table I.

The peak CFU-GM mL$^{-1}$ blood ($0.2–44.1 \times 10^3$) was inversely correlated to the number of days to reach a WBC of

**Figure 1** Kinetics of peripheral blood CFU-GM in relation to WBC **a**, and platelet recovery **b**, after Dexa-BEAM ($n = 11$). The ordinate depicts the medians of the CFU-GM percentages on the corresponding days relative to the individual CFU-GM maxima ( = 100%).

$>10\,nL^{-1}$ (rs = − 0.68, $P<0.025$) and to the number of days to reach a WBC of $>20\,nL^{-1}$ (rs = − 0.80, $P<0.005$). Among the patients for whom PBPC kinetics were available, three had strikingly low numbers of peripheral blood CFU-GM (peak CFU-GM $<1\times10^3\,mL^{-1}$); in all of them WBC recovery was slow and incomplete as indicated by the fact that a WBC of $>20\,nL^{-1}$ was not reached in spite of continued G-CSF application (Table II). All patients with 'normal' CFU-GM values in their peripheral blood achieved a WBC of $>10\,nL^{-1}$ within 3 weeks from the start of Dexa-BEAM and exceeded a WBC of $20\,nL^{-1}$ 1 or 2 days later. A clear-cut relationship between PBPC maxima and the intensity of pretreatment was not evident.

*CD34-positive cells*

In the first three patients, labelling of CD34-positive cells was performed with QBEND10-FITC. However, as the fluorescence intensity obtained with this conjugate was found to be very low, a reliable discrimination of CD34 + cells was not possible. Thus, in all subsequent patients, CD34 + cells were also stained with 8G12-PE. This antibody, which binds to a chymopapain-resistant epitope on the CD34 molecule (Lansdorp *et al.*, 1989; Civin *et al.*, 1990), produces a bright specific fluorescence, allowing the reproducible quantitation of even very small amounts of peripheral blood CD34 + MNC (>0.1% of gated cells). In our study, the CD34 +

**Table II** Kinetics of PBPC mobilisation and WBC recovery after Dexa-BEAM

| *UPN* | *WBC >0.5 nL⁻¹ day* | *WBC >10 nL⁻¹ day* | *WBC >20 nL⁻¹ day* | *Platelets >50 nL⁻¹ day* | *CFU-max day* | *CFU max × 10³ mL⁻¹ blood* | *CD34 max × 10³ mL⁻¹ blood* |
|---|---|---|---|---|---|---|---|
| P03 | 17 | 19 | 20 | na[a] | na | na | na |
| P04 | 19 | 21 | 23 | 21 | 22 | 7.2 | na |
| P05 | na | 19 | na | na | na | na | na |
| P06 | 22 | 28 | nr[b] | 27 | 28 | 0.52 | 9.3 |
| P07 | 20 | 23 | nr | 25 | 24 | 0.2 | 3.4 |
| P08 | 17 | 20 | 21 | 21 | 21 | 3.19 | 49.5 |
| P09 | 16 | 18 | 20 | 18 | 19 | 44.06 | 296.6 |
| P12 | na | na | na | na | na | na | na |
| P13 | 19 | 22 | 23 | 21 | 22 | 11.25 | 166.7 |
| P14 | 18 | 20 | 21 | na | na | na | na |
| P15 | 17 | 19 | 19 | 17 | 20 | 31.56 | 258 |
| P17 | 17 | 18 | 19 | 19 | 19 | 8.59 | 93 |
| P18 | 19 | 21 | nr | 20 | 24 | 0.5 | 9.1 |
| P19 | 18 | 20 | 21 | 21 | 20 | 4.37 | 26.3 |
| P20 | 17 | 19 | 21 | 20 | 21 | 8.36 | 42.8 |
| Median | 18 | 20 | 21 | 21 | 21 | 7.2 | 46.2 |
| Range | 16-22 | 18-28 | 19->28 | 17-27 | 19-28 | 0.2-44.06 | 3.4-296.6 |
| *n* | 13 | 14 | 13 | 11 | 11 | 11 | 10 |

[a]Not available. [b]Not reached.

MNC populations demonstrated almost entirely low right-angle light scatter and low to intermediate forward light scatter characteristics, corresponding to the 'lymphocyte window' as defined by SIENA *et al.* (Siena *et al.*, 1989) (Figure 2).

When measured with 8G12-PE, the kinetics of peripheral blood CD34 brightly positive cells paralleled those of CFU-GM very closely ($r = 0.90$); the relation might be fitted by a linear regression line described by the equation $y = 0.084 + 0.102x$, in which x corresponds to the CD34 + cell count and y to the CFU-GM count. The slope of the regression line indicates that CFU-GM were present at 0.102 the number of CD34 + cells, or, vice versa, CD34 + cells were 9.8 times more frequent than CFU-GM. Considering each individual pair of samples, five to 20 CD34 + cells were found per one CFU-GM. The maximum percentage of CD34 + cells ranged from 0.16 to 4.21% (median 2.22%) of MNC, according to absolute numbers of $3.4-296.6 \times 10^3$ CD34 + cells per mL blood. In patient P07, who displayed the lowest numbers of PBPC, flow cytometry yielded 0.04–0.16% CD34 + cells without clear correlation to the CFU-GM counts, indicating a limited precision of CD34 + cell quantitation when very small percentages of CD34 + cells are present. (This patient was not included in the analysis depicted in Figure 3).

Linear regression analysis of CFU-GM $\times 10^4$/CD34 + cells $\times 10^5$ in the harvests of 25 leukapheresis procedures again revealed a strong correlation between these two parameters ($r = 0.95$; $y = 8.13 + 0.784x$, corresponding to 12.8 CD34 + cells per CFU-GM). The correlation between BFU-E and CD34 + cells was less striking ($r = 0.75$; $y = 2.55 + 0.255x$, corresponding to 39.2 CD34 + cells per BFU-E; Figure 3).

### *Composition of the leukapheresis products*

Overall, 2–6 leukapheresis procedures yielded $1.82-13.49 \times 10^8$ MNC $kg^{-1}$ (median 6.39), containing $2.2-213.8 \times 10^4$ CFU-GM $kg^{-1}$ (median 44.4), $4.8-101.1 \times 10^4$ BFU-E $kg^{-1}$ (median 26.8), and $0.14-4.52 \times 10^8$ lymphocytes (median 1.31, Table III). When performing an immunophenotypical analysis on the leukapheresis products of 9 patients, we found $16.2-269.7 \times 10^5$ CD34 + cells $kg^{-1}$ (median 57), $28-345.9 \times 10^6$ CD31 + T cells $kg^{-1}$ (median 116), 20.5–

**Figure 2** Flow cytometry of CD34 + cells in the peripheral blood of patients treated with Dexa-BEAM + G-CSF. Shown are examples of bivariate plots displaying right-angle scatter properties *vs* staining with 8G12-PE (right panels) or irrelevant mouse IgG-PE (left panels). Calculation windows include 0 *vs* 0.20 (UPN P18), and 0 *vs* 1.90% PE-postive cells (UPN P20).

251.2 × $10^6$ CD4 + T cells $kg^{-1}$ (median 59.7), 7.1–100.4 × $10^6$ CD8 + T cells $kg^{-1}$ (median 53.6), and 2.6–98 × $10^6$ CD56 + CD3- NK cells $kg^{-1}$ (median 23.8). The percentage of CD19 + B cells was remarkably low in all collection products (usually less than 1%), resulting in a B cell content of 0–5.9 × $10^6$ cells $kg^{-1}$ (median 0.7).

*Engraftment after PBPC reinfusion*

Nine patients have been autografted with PBPC (15.4–213.8 × $10^4$ CFU-GM $kg^{-1}$) after myeloablative chemotherapy ('CVB' or 'BEAM' regimen). All experienced rapid and sustained engraftment (neutrophils $>0.5\ nL^{-1}$ on day 8–14 [median 10], untransfused platelets $>20\ nL^{-1}$ on day 7–14 [median 10], and platelets $>50\ nL^{-1}$ on day 9–22 [median 13]) (Table IV). One patient was given a PBPC graft containing 4.8 × $10^4$ CFU-GM $kg^{-1}$, and, in addition, autologous marrow containing 3.5 × $10^4$ CFU-GM $kg^{-1}$. Although receiving stem cells from both sources, this patient engrafted relatively slow; in particular, the normalisation of the platelet count was delayed (platelets > 50 on day + 199). Overall, the amount of CFU-GM infused showed an inverse correlation to the number of days needed to achieve an unsupported platelet count of $>20\ nL^{-1}$ (rs = − 0.73, $P<0.02$) or $>50\ nL^{-1}$ (rs = − 0.74, $P<0.01$, Spearman's rank correlation test).

**Discussion**

The data presented here demonstrate that PBPC can be effectively mobilised by salvage combination chemotherapy (Dexa-BEAM) plus G-CSF. Although it has been recommended that leukapheresis after cytotoxic mobilisation should start as soon as WBC have reached 1 $nL^{-1}$ (Gianni *et al.*, 1989; Siena *et al.*, 1989; Emminger *et al.*, 1990), and this practice has been adapted for combined cytotoxic/CSF-mediated mobilisation (Siena *et al.*, 1991; Teshima *et al.*, 1992), our experience indicates that the optimum time for harvesting PBPC after Dexa-BEAM + G-CSF is not before the WBC has increased to more than 10 $nL^{-1}$. If at this time leukapheresis is not possible, sufficient amounts of PBPC can still be collected on at least five subsequent days, provided the G-CSF administration is continued. The absolute numbers of PBPC present in the circulation correlated well with the speed and completeness of WBC recovery, i.e. individuals who exhibited a leukocyte count of 10 $nL^{-1}$ relatively late and never reached 20 $nL^{-1}$ displayed CFU-GM maxima that were 10–100 times lower than those seen in patients with a rapid WBC recovery to $>20\ nL^{-1}$. This observation matches findings obtained in cytotoxic mobilisation (Emminger *et al.*, 1990).

As reported for other mobilisation regimens, CFU-GM maxima were also associated with platelet recovery after

**Figure 3** Correlations between CD34 + cells and **a**, CFU-GM in 57 peripheral blood samples from 12 patients, **b**, CFU-GM in 25 leukapheresis products from nine patients, and **c**, BFU-E in 25 leukaphereis products from nine patients. Calculations were done using linear regression analysis, for clarity, plots are given on a logarithmic scale.

**Table III** Composition of the leukapheresis products

| *UPN* | *No. of aph*[a] | *CD34* × $10^5$ $kg^{-1}$ | *CFU-GM* × $10^4$ $kg^{-1}$ | *BFU-E* × $10^4$ $kg^{-1}$ | *MNC* × $10^8$ $kg^{-1}$ | *Lymphoc.* × $10^8$ $kg^{-1}$ |
|---|---|---|---|---|---|---|
| P03 | 6 | na | 4.8 | na | 13.49 | na |
| P04 | 4 | na | 50.5 | 14.2 | 4.09 | 1.51 |
| P05 | 4 | na | 15.2 | 6.2 | 5.84 | 0.21 |
| P06 | 4 | na | 2.2 | 4.8 | 1.82 | 0.14 |
| P07 | 4 | na | 3.5 | na | 4.99 | 0.45 |
| P08 | 4 | na | 43.3 | 12.6 | 8.66 | 1.41 |
| P09 | 3 | 175.7 | 203.2 | 33 | 10.35 | 1.19 |
| P12 | 3 | 57 | 65.8 | 29.5 | 8.17 | 4.52 |
| P13 | 3 | 269.7 | 206.2 | 32.7 | 8.17 | 1.51 |
| P14 | 3 | 24.4 | 19.4 | 10.9 | 7.06 | 2.4 |
| P15 | 2 | 245.8 | 213.8 | 101.1 | 9.81 | 1.71 |
| P17 | 2 | 54.9 | 44.4 | 26.8 | 6.39 | 1.2 |
| P18 | 3 | 16.2 | 12 | 9.1 | 3.74 | 1.02 |
| P19 | 3 | 35.2 | 65.8 | 27.9 | 5.58 | 0.31 |
| P20 | 3 | 62.6 | 110.3 | 32.8 | 5.47 | 1.79 |
| Median | 3 | 57 | 44.4 | 26.8 | 6.39 | 1.31 |
| Range | 2–6 | 16.2–269.7 | 2.2–213.8 | 4.8–101.1 | 1.82–13.49 | 0.14–4.52 |
| *n* | 15 | 9 | 15 | 13 | 15 | 14 |

[a]Number of leukaphereses

**Table IV** Kinetics of engraftment after PBPC reinfusion

| UPN | *CFU-GM infused* ( $\times 10^4$ $kg^{-1}$) | *Neutrophils* $> 0.5$ $nL^{-1}$ *(day)* | *Platelets* $> 20$ $nL^{-1}$ *(day)* | *Platelets* $> 50$ $nL^{-1}$ *(day)* | *Hosp. (days)* |
|---|---|---|---|---|---|
| P03 | 4.8[a] | + 10 | + 20 | + 199 | 19 |
| P04 | 50.5 | + 10 | + 12 | + 18 | 16 |
| P05 | 15.2[b] | + 10 | + 10 | + 14 | 14 |
| P06 | 22.6[c] | + 14 | + 14 | + 22 | 23 |
| P08 | 43.3[b] | + 13 | + 10 | + 13 | 14 |
| P09 | 203.2 | + 8 | + 8 | + 9 | 11 |
| P12 | 65.8[b] | + 11 | + 10 | + 10 | 16 |
| P13 | 206.2 | + 9 | + 10 | + 13 | 21 |
| P14 | 19.4 | + 10 | + 11 | + 16 | 18 |
| P15 | 213.8 | + 8 | + 7 | + 11 | 15 |
| Median | 46.9 | 10 | 10 | 13 | 16 |
| Range | 4.8–213.8 | 8–14 | 7–20 | 9–199 | 11–23 |

[a]Autologous BM containing $3.52 \times 10^4$ CFU-GM $kg^{-1}$ was administered simultaneously. [b]No G-CSF after PBPC infusion. [c]Including $20.4 \times 10^4$ G-CSF-mobilised CFU-GM $kg^{-1}$ obtained on a separate occasion.

Dexa-BEAM (Teshima *et al.*, 1992; Emminger *et al.*, 1990). However, with regard to timing of leukapheresis, the leukocyte count appears to be the more suitable indicator of PBPC expansion because WBC recovery usually becomes evident 2–3 days before the critical value (10 $nL^{-1}$) is reached, whereas the platelet rise often is obscured by platelet transfusions. Moreover, CFU-GM peaks were always preceded by achievement of the critical leukocyte value (10 $nL^{-1}$) but not of the critical platelet value (50 $nL^{-1}$).

While it is well documented that the addition of growth factors strongly increases the efficacy of cytotoxic PBPC mobilisation, investigations indicating the augmentation of CSF-mediated mobilisation by cytotoxic therapy are sparse. Accordingly, in our protocol the importance of Dexa-BEAM for expanding the PBPC pool is not clearly defined. However, the relevance of chemotherapy in this setting is underlined by the fact that we were not able to collect $100 \times 10^4$ CFU-GM $kg^{-1}$ or more with 2–3 leukapheresis in a limited number of patients mobilised with G-CSF alone, although previous treatment in general was less intensive than in the cohort treated with Dexa-BEAM + G-CSF (unpublished observations). Larger patient numbers, however, are required to draw definite conclusions on this topic.

The number of CFU-GM necessary for successful trilineage engraftment has been a subject of controversy; recommended minimum CFU-GM doses range from $10 \times 10^4$ to $50 \times 10^4$ $kg^{-1}$ (Siena *et al.*, 1991; Teshima *et al.*, 1992; To *et al.*, 1986; To & Juttner, 1987). This discrepancy may reflect differences in the CFU-GM assays employed, in the agents used for mobilisation, and in the underlying diseases. In our series, rapid and complete engraftment was achieved in each patient who received $10 \times 10^4$ CFU-GM $kg^{-1}$ or more; the single patient transplanted with $4.8 \times 10^4$ CFU-GM $kg^{-1}$ showed delayed platelet recovery in spite of simultaneous administration of autologous bone marrow. Thus, fewer than $10 \times 10^4$ CFU-GM $kg^{-1}$ (or $20 \times 10^5$ CD34 + cells $kg^{-1}$) would appear insufficient to ensure complete engraftment in this setting. Altogether, engraftment after transplantation of Dexa-BEAM + G-CSF-mobilised PBPC compares favourably with the recovery of 29 historical control patients with lymphoma grafted with autologous bone marrow (platelets $> 20$ $nL^{-1}$ on day 16–143 [median 25], platelets $> 50$ $nL^{-1}$ on day 20–235 [median 31], manuscript in preparation).

The relationship between CD34 and CFU-GM in peripheral blood is well-acknowledged (Bender *et al.*, 1992; Siena *et al.*, 1989; Siena *et al.*, 1991; Brugger *et al.*, 1992; Matsunaga *et al.*, 1993). However, the correlation found in the present series is much stronger than in most previous studies, which reported correlations between CD34 and CFU-GM $mL^{-1}$ ranging from $r = 0$ to $r = 0.89$ (Bender *et al.*, 1992; Siena *et al.*, 1989; Siena *et al.*, 1991; Brugger *et al.*, 1992; Matsunaga *et al.*, 1993; Janssen *et al.*, 1992; Haas *et al.*, 1992). This may have various reasons: (1) As we and others have observed (Lansdorp *et al.*, 1989), the affinity and the degree of unspecific binding between different CD34 MoAbs and conjugates varies considerably. A less discriminating CD34 conjugate or indirect CD34 labelling may include unspecific fluorescence and thus affect the CD34/CFU-GM correlation (Brugger *et al.*, 1992; Janssen *et al.*, 1992; Haas *et al.*, 1992). On the contrary, 8G12-PE produces an intensive staining of CD34 + cells with low right-angle light scatter and low to intermediate forward light scatter characteristics, allowing a narrow gating on bright fluorescent cells that excludes unspecific fluorescence and dim CD34 + cells. Using this technique, a clear-cut identification and reproducible quantification of even small proportions of CD34 + cells is possible. Probably due to the exclusion of cells of uncertain specificity and dim CD34 + cells, the relative and absolute numbers of CD34 + cells were lower on a per mL and per CFU-GM basis in this study than in others in which an indirect CD34-labelling was employed (Siena *et al.*, 1989; Matsunaga *et al.*, 1993), but similar to the results obtained by SIENA *et al.* who used a 8G12-FITC conjugate (Siena *et al.*, 1991). (2) The fact that colony counting was performed by the same person throughout the whole study may have contributed to the strong CD34 + cell/CFU-GM correlation observed. (3) Finally, the degree of correlation between CFU-GM and CD34 + cells may depend on the method of PBPC mobilisation (Brugger *et al.*, 1992; Janssen *et al.*, 1992). Altogether, in our study, the CD34 + cell/CFU-GM ratios ranged consistently between 20:1 and 5:1, implying that a leukapheresis yield of $> 20 \times 10^5$ CD34 + cells $kg^{-1}$ reliably predicted for a CFU-GM content of $> 10 \times 10^4$ $kg^{-1}$.

When analysing the cellular components of the collection products by immunophenotyping, we found that CD19 + B cells were strongly reduced or undetectable in the PBPC grafts. This phenomenon was much more pronounced in the present series than observed after cytotoxic mobilisation with other agents (Kiesel *et al.*, 1989), and paralleled findings obtained after BM transplantation (BMT), where B cells have been reported to be virtually absent from the peripheral blood in the first few weeks after transplant (Ault *et al.*, 1985; Aotsuka *et al.*, 1991; Dreger *et al.*, 1993*b*). Thus, Dexa-BEAM appears to produce some kind of '*in vivo* B cell depletion' of PBPC grafts. Since PBPCT is used in patients with B cell NHL with BM involvement (Kessinger *et al.*, 1989; Hardingham *et al.*, 1993), this finding is of particular importance because Dexa-BEAM plus G-CSF might be employed to 'purge' autologous stem cell grafts of such patients.

The NK cell/T cell ratios in the PBPC grafts were in the range expected for normal blood and different from those seen early after BMT, where the NK cell compartment is increased (Ault *et al.*, 1985; Aotsuka *et al.*, 1991). The PBPC grafts contained a median T cell dose of $1.16 \times 10^8$ $kg^{-1}$, or at least three times more T cells than an average BM graft (Mitsuyasu *et al.*, 1986). Given that T cells play a role in engraftment of allogeneic and autologous BM (Martin,

1990), the high T cell dose contaminating PBPC grafts may contribute to the fast hematopoietic recovery after PBPCT. On the other hand, the high T cell content of PBPC grafts might be a major obstacle for the use of G-CSF-mobilised PBPC in the allogeneic setting (Dreger *et al.*, 1993*a*), where T cells are thought to represent the main carriers of graft-versus-host reactivity.

Taken together, Dexa-BEAM plus G-CSF can mobilise large amounts of PBPC that are capable of mediating rapid and sustained haematopoietic recovery after high-dose chemotherapy. The progenitor cell content of the leukapheresis produce correlates with the speed of leukocyte recovery after Dexa-BEAM and can rapidly and reliably be assayed by measurement of 8G12-PE (CD34) brightly positive cells. From a practical point of view, PBPC collection should start immediately after the WBC has exceeded $10.0\,nL^{-1}$ and might be terminated as soon as more than $20 \times 10^5$ CD34 + cells are harvested. This schedule (which should apply also to patients treated with the very similar mini-BEAM protocol) will allow asservation of autologous stem cells with a minimum number of leukaphereses, minimum costs, and without causing delay in continuation of salvage chemotherapy.

We thank the staff of the dialysis, haematology and bone marrow transplantation wards for excellent patient care and cooperation. The technical assistance of Ms Susanne Holst and Ina Wertz is gratefully acknowledged.

### References

AOTSUKA, N., ASAI, T., OH, H., YOSHIDA, S., ITOH, K. & SATO, T. (1991). Lymphocyte subset reconstitution following human allogeneic bone marrow transplantation: differences between engrafted patients and graft failure patients. *Bone Marrow Transplantation*, **8**, 345–349.

AULT, K.A., ANTIN, J.H., GINSBURG, D., ORKIN, S.H., RAPPEPORT, J.M., KEOHAN, M.L., MARTIN P. & SMITH, B.R. (1985). Phenotype of recovering lymphoid cell populations after marrow transplantation. *J. Exp. Med.*, **161**, 1483–1502.

BENDER, J.G., WILLIAMS, S.F,. MYERS, S., NOTTLEMAN, D., LEE, W.J., UNVERZAGT, K.L., WALKER, D., TO, L.B. & VAN EPPS, D.E. (1992). Characterization of chemotherapy mobilized peripheral blood progenitor cells for use in autologous stem cell transplantation. *Bone Marrow Transplantation*, **10**, 281–285.

BRANDWEIN, J.M., SMITH, A.M., LANGLEY, G.R., BURNELL, M., SUTCLIFFE, S.B. & KEATING, A. (1991). Outcome of patients with relapsed or refractory Non-Hodgkin's lymphoma referred for autologous bone marrow transplantation. *Leukemia & Lymphoma*, **4**, 231–238.

BRUGGER, W., BROSS, K., FRISCH, J., DERN, P., WEBER, B., MERTELSMANN, R. & KANZ, L. (1992). Mobilization of peripheral blood progenitor cells by sequential administration of interleukin-3 and granulocyte-macrophage colony-stimulating factor following polychemotherapy with Etoposide, Ifosfamide, and Cisplatin. *Blood*, **79**, 1193–1200.

CHOPRA, R., LINCH, D.C., MCMILLAN, A.K., BLAIR, S., PATTERSON, K.G., MOIR, D., RICHARDS, J.D.M., CERVI, P., KINSEY, S. & GOLDSTONE, A.H. (1992). Mini-BEAM followed by BEAM and ABMT for very poor risk Hodgkin's disease. *Br. J. Haematol.*, **81**, 197–202.

CIVIN, C.I., STRAUSS, L.C., FACKLER, M.J., TRISCHMANN, T.M., WILEY, J.M. & LOKEN, M.R. (1990). Positive stem cell selection – basic science. *Prog. Clin. Biol. Res.*, **33**, 387–402.

DELUCA, E., SHERIDAN, W.P., WATSON, D., SZER, J. & BEGLEY, C.G. (1992). Prior chemotherapy does not prevent effective mobilisation by G-CSF of peripheral blood progenitor cells. *Br. J. Cancer*, **66**, 893–899.

DREGER, P., SUTTORP, M., HAFERLACH, T., SCHROYENS, W., LÖFFLER, H. & SCHMITZ, N. (1993*a*). Allogeneic G-CSF-mobilised peripheral blood progenitor cells for treatment of engraftment failure after bone marrow transplantation. *Blood*, **81**, 1404–1407.

DREGER, P., GRELLE, K., ECKSTEIN, V., SUTTORP, M., MÜLLER-RUCHHOLTZ, W., LÖFFLER, H. & SCHMITZ, N. (1993*b*). Granulocyte-colony-stimulating factor induces increased serum levels of soluble interleukin 2 receptors preceding engraftment in autologous bone marrow transplantation. *Br. J. Haematol.*, **83**, 7–13.

DÜHRSEN, U., VILLEVAL, J.-L., BOYD, J., KANNOURAKIS, G., MORSTYN, G. & METCALF, D. (1988). Effects of recombinant human granulocyte colony-stimulating factor on hematopoietic progenitor cells in cancer patients. *Blood*, **72**, 2074–2081.

EMMINGER, W., EMMINGER-SCHMIDMEIER, W., HÖCKER, P., GERHARTL, C., KUNDI, M. & GADNER, H. (1990). The median daily increment of leukocytes during hematopoietic recovery reflects the myeloid progenitor cell yield during leukapheresis in children. *Bone Marrow Transplantation*, **5**, 419–424.

GIANNI, A.M., BREGNI, M., STERN, A.C., SIENA, S., TARELLA, C., PILERI, A. & BONADONNA, G. (1989). Granulocyte-macrophage colony-stimulating factor to harvest circulating haemopoietic stem cells for autotransplantation. *The Lancet*, **ii**, 580–585.

HAAS, R., HOHAUS, S., EGERER, G., EHRHARDT, R., WITT, B. & HUNSTEIN, W. (1992). Recombinant human granulocyte-macrophage colony-stimulating factor (rhGM-CSF) subsequent to chemotherapy improves collection of blood stem cells for autografting in patients not eligible for bone marrow harvest. *Bone Marrow Transplantation*, **9**, 459–465.

HARDINGHAM, J.E., KOTASEK, D., SAGE, R.E., DOBROVIC, A., GOOLEY, T. & DALE, B.M. (1993). Molecular detection of residual lymphoma cells in peripheral blood stem cell harvests and following autologous transplantation. *Bone Marrow Transplantation*, **11**, 15–20.

JANSSEN, W.E., FARMELO, M.J., LEE, C., SMILEE, R., KRONISH, L. & ELFENBEIN, G.J. (1992). The CD34 + cell fraction in bone marrow and blood is not universally predictive of CFU-GM. *Exp. Hematol.*, **20**, 528–530.

KESSINGER, A., ARMITAGE, J.O., SMITH, D.M., LANDMARK, J.D., BIERMAN, P.J. & WEISENBURGER, D.D. (1989). High-dose therapy and autologous peripheral blood stem cell transplantation for patients with lymphoma. *Blood*, **74**, 1260–1265.

KIESEL, S., PEZZUTTO, A., KÖRBLING, M., HAAS, R,. SCHULZ, R., HUNSTEIN, W. & DÖRKEN, B. (1989). Autologous peripheral blood stem cell transplantation: analysis of autografted cells and lymphocyte recovery. *Transplantation Proceedings*, **21**, 3084–3088.

LANSDORP, P.M., DOUGHERTY, G.J. HUMPHRIES, R.K. (1989). CD34 epitopes. *Leucocyte Typing IV* by Knapp, W., Dörken, B., Rieber, E.P., Stein, H., Gilks, W.R., Schmidt, R.E. & Von Dem Borne, A.E.G.K. (eds.) p. 826. Oxford University Press, Oxford.

MARTIN, P.J. (1990). The role of donor lymphoid cells in allogeneic marrow engraftment. *Bone Marrow Transplantation*, **6**, 283–289.

MATSUNAGA, T., SAKAMAKI, S., KOHGO, Y,. OHI, S., HIRAYAMA, Y. & NIITSU, Y. (1993). Recombinant human granulocyte colony-stimulating factor can mobilize sufficient amounts of peripheral blood stem cells in healthy volunteers for allogeneic transplantation. *Bone Marrow Transplantation*, **11**, 103–108.

MITSUYASU, R.T., CHAMPLIN, R.E., GALE, R.P., HO, W.G., LENARSKY, C., WINSTON, D., SELCH, M., ELASHOFF, R., GIORGI, J.V., WELLS, J., TERASAKI, P., BILLING, R. & FEIG, S. (1986). Treatment of donor bone marrow with monoclonal anti-T-cell antibody and complement for the prevention of graft-versus-host disease. *Ann. Int. Med.*, **105**, 20–26.

PFREUNDSCHUH, M., RUEFFER, U., LATHAN, B., SCHMITZ, N., BROSTEANU, O., HASENCLEVER, D., HAAS, R., KOCH, P., KUSE, R., LOEFFLER, M. & DIEHL, V. (1993). Dexa-BEAM in patients with Hodgkin's disease refractory to multi-drug regimens: a multicenter trial of the German Hodgkin's Study Group. (submitted).

PHILIP, T., ARMITAGE, J.O., SPITZER, G., CHAUVIN, F., JAGANNATH, S., CAHN, J.-Y., COLOMBAT, P., GOLDSTONE, A.H., GORIN, N.C., FLESH, M., LAPORTE, J.-P., MARANINCHI, D., PICO, J., BOSLY, A., ANDERSON, C., SCHOTS, R., BIRON, P., CABANILLAS, F. & DICKE, K. (1987). High-dose therapy and autologous bone marrow transplantation after failure of conventional chemotherapy in adults with intermediate-grade or high-grade non-Hodgkin's lymphoma. *New England J. Med.*, **316**, 1493–1498.

RICHMAN, C.M., WEINER, R.S. & YANKEE, R.A. (1976). Increase in circulating stem cells following chemotherapy in man. *Blood*, **47**, 1031–1039.

SCHMITZ, N., GASSMANN, W., RISTER, M., JOHANNSON, W., SUTTORP, M., BRIX, F., HOLTHUIS, J.J.M., HEIT, W., HERTENSTEIN, B., SCHAUB, J. & LÖFFLER, H. (1988). Fractionated total body irradiation and high-dose VP 16-213 followed by allogeneic bone marrow transplantation in advanced leukemias. *Blood*, **72**, 1567–1573.

SCHMITZ, N., GLASS, B., DREGER, P., HAFERLACH, T., HORST, H.-A., OLLECH-CHWOYKA, J., SUTTORP, M., GASSMANN, W. & LÖFFLER, H. (1993). High-dose chemotherapy and hematopoietic stem cell rescue in patients with relapsed Hodgkin's disease. *Ann. Hematol.*, **66**, 251–256.

SHERIDAN, W.P., BEGLEY, C.G., JUTTNER, C.A., SZER, J., TO, L.B., MAHER, D., MCGRATH, K.M., MORSTYN, G. & FOX, R.M. (1992). Effect of peripheral-blood progenitor cells mobilized by filgrastim (G-CSF) on platelet recovery after high-dose chemotherapy. *The Lancet*, **339**, 640–644.

SIENA, S., BREGNI, M., BRANDO, B., RAVAGNANI, F, BONADONNA, G. & GIANNI, A.M. (1989). Circulation of CD34+ hematopoietic stem cells in the peripheral blood of high-dose cyclophosphamide-treated patients: enhancement by intravenous recombinant human granulocyte-macrophage colony-stimulating factor. *Blood*, **74**, 1905–1914.

SIENA, S., BREGNI, M., BRANDO, B., BELLI, N., RAVAGNANI, F., GANDOLA, L., STERN, A.C., LANSDORP, P.M., BONADONNA, G. & GIANNI, A.M. (1991). Flow cytometery for clinical estimation of circulating hematopoietic progenitors for autologous transplantation in cancer patients. *Blood*, **77**, 400–409.

SOCINSKI, M.A., ELIAS, A., SCHNIPPER, L., CANNISTRA, S.A., ANTMAN, K.H. & GRIFFIN, J.D. (1988). Granulocyte-macrophage colony stimulating factor expands the circulating haemopoietic progenitor cell compartment in man. *The Lancet*, **ii**, 1194–1198.

STEWART, A.K., BRANDWEIN, J.M., SUTCLIFFE, S.B., SCOTT, J.G. & KEATING, A. (1991). Mini-beam as salvage chemotherapy for refractory Hodgkin's disease and non-Hodgkin's lymphoma. *Leukemia & Lymphoma*, **5**, 111–115.

TESHIMA, T., HARADA, M., TAKAMATSU, Y,. MAKINO, K,. TANIGUCHI, S., INABA, S., KONDO, S., TANAKA, T., AKASHI, K., MINAMISHIMA, I., ISHII, E., NISHIMURA, J. & NIHO, Y. (1992). Cytotoxic drug and cytotoxic drug/G-CSF mobilisation of peripheral blood stem cells and their use for autografting. *Bone Marrow Transplantation*, **10**, 215–220.

TO, L.B., DYSON, P.G. & JUTTNER, C.A. (1986). Cell-dose effect in circulating stem-cell autografting. *The Lancet*, **ii**, 404–405.

TO, L.B., DAVY, M.L.J., HAYLOCK, D.N., DYSON, P.G., THORP, D. & JUTTNER, C.A. (1989). Autotransplantation using peripheral blood stem cells mobilized by cyclophosphamide. *Bone Marrow Transplantation*, **4**, 595–596.

TO, L.B. & JUTTNER, C.A. (1987). Annotation. Peripheral blood stem cell autografting: a new therapeutic option for AML? *Br. J. Haematol.*, **66**, 285–288.

TO, L.B., ROBERTS, M.M., HAYLOCK, D.N., DYSON, P.G., BRANFORD, A.L., THORP, D., HO, J.Q.K., DART, G.W., HORVATH, N., DAVY, M.L.J., OLWENY, C.L.M., ABDI, E. & JUTTNER, C.A. (1992). Comparison of haematological recovery times and supportive care requirements of autologous recovery phase peripheral blood stem cell transplants, autologous bone marrow transplants and allogeneic bone marrow transplants. *Bone Marrow Transplantation*, **9**, 277–284.